# Antinuclear Antibodies Predict Treatment Escalation and Biologic Switching in Rheumatoid Arthritis

**DOI:** 10.3390/diagnostics16060957

**Published:** 2026-03-23

**Authors:** Zeynel Abidin Akar, Dilan Yıldırım, Mehmet Çiftçi, Zeynep Işık Sula, Serap Karaman, Remzi Çevik, Mehmet Karakoç, Serda Em, İbrahim Batmaz, Pelin Oktayoğlu, Mehmet Çağlayan

**Affiliations:** 1 Division of Rheumatology, Department of Physical Therapy and Rehabilitation, Faculty of Medicine, Dicle University, 21280 Diyarbakır, Türkiye; 2 Department of Physical Therapy and Rehabilitation, Faculty of Medicine, Dicle University, 21280 Diyarbakır, Türkiye

**Keywords:** rheumatoid arthritis, antinuclear antibodies, biologic therapy, Cox regression, treatment escalation, prognostic biomarkers

## Abstract

**Background:** Antinuclear antibodies (ANAs) are frequently detected in patients with rheumatoid arthritis (RA); however, their prognostic relevance for predicting treatment escalation and biologic therapy initiation remains incompletely understood. Identifying biomarkers associated with earlier transition to advanced therapies may enhance individualized, treat-to-target disease management. **Objectives:** We aimed to evaluate the association of ANA status and titer levels with clinical characteristics, treatment trajectories, and time to biologic therapy initiation in patients with RA. **Methods:** In this retrospective cohort study, 223 patients with RA were stratified according to ANA status (112 ANA-positive, 111 ANA-negative). Baseline demographic data, disease activity (DAS28), and serological markers (RF, anti-CCP) were analyzed. Time to biologic therapy initiation, defined from the date of RA diagnosis to first biologic or targeted synthetic DMARD use, was assessed using Kaplan–Meier survival analysis and Cox proportional hazards regression. Multivariate models adjusted for clinically relevant covariates (age, sex, disease duration, RF, anti-CCP). Within the ANA-positive group, exploratory analyses compared low–moderate (1:80–1:320) and high (>1:320) ANA titers, highlighting potential non-linear effects. **Results:** Baseline demographic and clinical characteristics were comparable between groups (all *p* > 0.05). ANA-positive patients more frequently initiated biologic therapy (48.2% vs. 24.3%, *p* < 0.001) and experienced multiple biologic switches (29.5% vs. 16.2%, *p* = 0.028). In multivariate analysis, ANA positivity independently predicted earlier biologic therapy initiation (adjusted HR 2.14; 95% CI 1.32–3.46; *p* = 0.002), whereas RF and anti-CCP status were not significant predictors. Exploratory subgroup analysis revealed the “titer paradox,” whereby high ANA titers (>1:320) were associated with a lower hazard of biologic therapy initiation compared with low–moderate titers (HR 0.24; 95% CI 0.06–0.98; *p* = 0.048). **Conclusions:** ANA positivity serves as an independent prognostic marker for earlier biologic therapy initiation in RA, providing incremental information beyond traditional serological markers. The observed non-linear association between ANA titers and treatment escalation underscores the need for cautious interpretation and validation in prospective, mechanistic studies, and highlights the potential value of integrating ANA profiling into personalized treatment strategies.

## 1. Introduction

Rheumatoid arthritis (RA) is a chronic, systemic autoimmune disease characterized by persistent synovial inflammation that may lead to progressive joint destruction, functional impairment, and reduced quality of life if inadequately controlled [1]. Over the past two decades, the implementation of treat-to-target strategies has substantially improved clinical outcomes [2]. In parallel, the expansion of available therapies—including conventional synthetic disease-modifying antirheumatic drugs (csDMARDs), biologic DMARDs (bDMARDs), and targeted synthetic DMARDs (tsDMARDs)—has further enhanced disease control and long-term prognosis [3]. Nevertheless, therapeutic response remains heterogeneous [4], and a considerable proportion of patients require treatment escalation or switching due to inadequate response or loss of efficacy [5].

Serological biomarkers play a central role in RA diagnosis and prognostication [6]. Rheumatoid factor (RF) and anti–cyclic citrullinated peptide (anti-CCP) antibodies are well-established markers associated with disease classification and structural progression [7]. However, these antibodies do not fully explain variability in treatment response or the need for advanced therapies [8]. Antinuclear antibodies (ANA), although traditionally associated with other systemic autoimmune rheumatic diseases, are also detected in a substantial subset of patients with RA [9], with reported prevalence ranging from 15% to over 30% depending on the population studied and laboratory methodology [10].

However, reported ANA prevalence in RA cohorts varies considerably across studies, with some investigations describing higher frequencies depending on patient selection, referral patterns, and serological testing methodology. For example, studies conducted in tertiary referral centers or using highly sensitive indirect immunofluorescence assays on HEp-2 cells have occasionally reported ANA positivity rates approaching 40% or higher in RA populations. These differences highlight the importance of considering methodological and population-related factors when interpreting ANA prevalence in RA cohorts [9,10,11].

The clinical significance of ANA in RA remains incompletely understood [9]. Some studies have suggested associations between ANA positivity and higher inflammatory burden, broader autoantibody repertoires, extra-articular manifestations, or features of polyautoimmunity [11]. Emerging immunologic data further indicate that subsets of RA characterized by distinct molecular signatures—such as enhanced type I interferon pathway activation—may exhibit differential clinical trajectories and therapeutic responses [12]. These observations raise the possibility that ANA positivity could reflect a distinct immunophenotype with implications for treatment outcomes [13].

Despite these considerations, most prior investigations have focused on cross-sectional associations between ANA and disease activity or correlations with classical serological markers [14]. Data examining the longitudinal relationship between ANA status and transition to advanced therapies remain limited [15]. In particular, whether ANA positivity independently predicts earlier initiation of biologic treatment—beyond established prognostic markers such as RF and anti-CCP—has not been clearly established [16]. Furthermore, the potential clinical relevance of ANA titer levels, rather than simple binary positivity, has been insufficiently explored [17].

In the context of precision medicine and treat-to-target management, identifying biomarkers associated with treatment escalation is of practical importance. Early recognition of patients at higher risk of inadequate response to csDMARDs may facilitate timely therapeutic adjustment and minimize prolonged exposure to uncontrolled inflammation [17].

Accordingly, the present study was designed to evaluate the association between ANA status and treatment trajectories in a well-characterized RA cohort. Specifically, we aimed to (1) compare baseline clinical characteristics according to ANA status, (2) assess the relationship between ANA positivity and time to biologic therapy initiation, adjusting for conventional serological markers, and (3) explore whether ANA titer levels are associated with differential treatment patterns. By addressing these questions, we sought to clarify the potential role of ANA as an adjunct prognostic biomarker in RA management.

## 2. Materials and Methods

### 2.1. Study Design and Ethical Approval

This retrospective cohort study was conducted at the Department of Rheumatology, Dicle University Faculty of Medicine. The study was performed in accordance with the ethical principles of the Declaration of Helsinki and was approved by the Dicle University Medical Faculty Ethics Committee for Non-Interventional Studies (Approval No: 62; 4 February 2026). Given the retrospective design and the use of anonymized clinical records, the requirement for written informed consent was waived.

### 2.2. Study Population

We screened adult patients (≥18 years) diagnosed with rheumatoid arthritis (RA) between January 2020 and December 2025. RA diagnosis was established according to the 2010 American College of Rheumatology/European Alliance of Associations for Rheumatology (ACR/EULAR) classification criteria [18]. Eligible patients were required to have documented antinuclear antibody (ANA) testing performed at the time of diagnosis and a minimum follow-up duration of 12 months. Patients were excluded if they had overlapping systemic autoimmune rheumatic diseases, including systemic lupus erythematosus, Sjögren’s syndrome, systemic sclerosis, or inflammatory myositis, in order to ensure that outcomes were attributable specifically to RA. Additional exclusion criteria included active malignancy at diagnosis or during follow-up, as well as incomplete baseline serological or longitudinal treatment data.

### 2.3. Data Collection and Baseline Variables

Demographic, clinical, and laboratory data were systematically retrieved from electronic medical records and standardized rheumatology follow-up forms. Baseline variables were defined at the time of RA diagnosis (time zero). Demographic characteristics included age, sex, body mass index (BMI), and smoking status.

Clinical variables comprised symptom duration prior to diagnosis, pattern of joint involvement, presence of extra-articular manifestations, and comorbidities such as hypertension, diabetes mellitus, and cardiovascular disease. Disease activity was assessed using the Disease Activity Score in 28 joints (DAS28). Systemic inflammation was evaluated through erythrocyte sedimentation rate (ESR) and C-reactive protein (CRP) levels.

### 2.4. Serological Assessment

ANA status was determined at diagnosis using indirect immunofluorescence (IIF) on HEp-2 cells in accordance with international consensus recommendations. A titer of ≥1:80 was considered positive.

All ANA analyses were performed in the central clinical immunology laboratory of our institution using a standardized HEp-2 indirect immunofluorescence platform. The positivity threshold (≥1:80) and laboratory protocol remained consistent throughout the study period, minimizing potential bias related to temporal changes in testing methodology. To assess the potential impact of antibody concentration, ANA-positive patients were stratified into low–moderate titer (1:80–1:320) and high-titer (>1:320) subgroups.

Rheumatoid factor (RF) and anti–cyclic citrullinated peptide (anti-CCP) antibodies were measured at diagnosis using standard laboratory techniques, including nephelometry and enzyme-linked immunosorbent assay (ELISA). All serological assessments were performed prior to the initiation of biologic or targeted synthetic therapies.

### 2.5. Treatment Exposure and Outcome Definition

Longitudinal treatment data were collected throughout the follow-up period and included the use of conventional synthetic DMARDs (csDMARDs), documentation of treatment failure, initiation of biologic DMARDs (bDMARDs), and initiation of targeted synthetic DMARDs (tsDMARDs). Biologic switching was defined as transition to a second or subsequent biologic agent during follow-up. Corticosteroid exposure was recorded as baseline dose at diagnosis, mean daily dose during follow-up, and cumulative dose. Cumulative corticosteroid exposure was calculated by summing all documented glucocorticoid doses recorded during follow-up and converting them to prednisone-equivalent doses to ensure consistency across different corticosteroid preparations.

The primary outcome was time to initiation of biologic or targeted synthetic DMARD therapy. Time zero was defined as the date of RA diagnosis. Time-to-event was calculated from diagnosis to the date of first biologic or targeted synthetic DMARD initiation. Patients who did not initiate biologic or targeted synthetic therapy during follow-up were censored at the date of their last documented clinical visit in the electronic medical record system.

### 2.6. Statistical Analysis

All statistical analyses were performed using SPSS Statistics version 27.0 (IBM Corp., Armonk, NY, USA). Continuous variables were expressed as mean ± standard deviation (SD) for normally distributed data or median with interquartile range (IQR) for non-normally distributed data, while categorical variables were presented as counts and percentages.

Normality was assessed using the Shapiro–Wilk or Kolmogorov–Smirnov test, as appropriate. Between-group comparisons were conducted using Student’s *t*-test or the Mann–Whitney U test for continuous variables and the Chi-square or Fisher’s exact test for categorical variables. All statistical tests were two-sided, and a *p*-value < 0.05 was considered statistically significant.

Time to biologic therapy initiation was evaluated using Kaplan–Meier survival analysis, and differences between groups were assessed using the log-rank test.

Univariate Cox proportional hazards regression analyses were performed to identify potential predictors of biologic therapy initiation.

Variables with *p* < 0.10 in univariate analysis, along with clinically relevant covariates (age, sex, symptom duration prior to diagnosis, RF positivity, anti-CCP positivity, and baseline corticosteroid dose), were entered into the multivariate Cox regression model.

Hazard ratios (HRs) and 95% confidence intervals (CIs) were calculated. The proportional hazards assumption was evaluated using Schoenfeld residuals and graphical inspection of log-minus-log survival plots, and no significant violations were detected.

An exploratory subgroup analysis was conducted within the ANA-positive cohort to evaluate differences in time to biologic therapy initiation according to ANA titer category (low–moderate versus high). Given the limited sample size of the high-titer subgroup, these analyses were considered hypothesis-generating.

The proportion of missing data was below 10% for all variables included in the analyses. Given the low degree of missingness, pairwise deletion was applied. Although no a priori sample size calculation was performed due to the retrospective design, post hoc assessment demonstrated adequate statistical power. Based on the observed event rate and hazard ratio estimates, the study had greater than 80% power to detect a hazard ratio of approximately 2.0 at a two-sided alpha level of 0.05. Additionally, the number of biologic therapy initiation events exceeded the recommended threshold of 10 events per variable included in the multivariate Cox model, supporting the stability and reliability of the regression estimates.

## 3. Results

The final analysis included 223 patients, of whom 112 were ANA-positive and 111 were ANA-negative. Baseline demographic and clinical characteristics are summarized in Table 1. Mean age was 54.2 ± 11.4 years in the ANA-positive group and 53.8 ± 12.1 years in the ANA-negative group (*p* = 0.78). Female predominance was 83.9% versus 80.1% (*p* = 0.45), and body mass index was similar across groups. Smoking status and major comorbidities, including hypertension, diabetes mellitus, and cardiovascular disease, did not differ significantly. Median disease duration was 114 months in ANA-positive patients and 108 months in ANA-negative patients (*p* = 0.61). Serological profiles, including RF and anti-CCP positivity, were also comparable (RF: 73.2% vs. 70.3%, *p* = 0.62; anti-CCP: 68.8% vs. 66.7%, *p* = 0.71). Baseline inflammatory markers (ESR, CRP) and disease activity scores (DAS28) did not significantly differ between groups, indicating comparable baseline clinical and serological profiles between ANA-positive and ANA-negative patients.

Distinct patterns in treatment escalation were observed according to ANA status (Table 2). ANA-positive patients were significantly more likely to initiate biologic therapy compared with ANA-negative patients (48.2% vs. 24.3%; *p* < 0.001). The median number of biologic agents used during follow-up was higher in ANA-positive patients (1.0 vs. 0.0; *p* = 0.004), and they were more likely to experience multiple biologic switches (≥2 agents) (29.5% vs. 16.2%; *p* = 0.028). It should be noted that biologic switching was descriptively reported to characterize treatment trajectories and was not analyzed as a primary outcome.

Differences were also observed in the choice of first-line biologic agents (*p* = 0.026). TNF inhibitors were the most frequently prescribed in both groups, while IL-6 inhibitors were more commonly used in the ANA-negative cohort (25.9% vs. 3.7%). The distribution of first biologic classes was calculated based on patients with available treatment classification data.

Corticosteroid-related parameters—including mean daily dose, cumulative dose, and total duration—were similar between groups. Conventional synthetic DMARD use and failure rates did not differ significantly.

Although the median time to biologic therapy initiation appeared numerically shorter in the ANA-negative group, this difference was not statistically significant in direct comparison. Time-to-event analyses using Kaplan–Meier curves and Cox proportional hazards modeling were therefore used as the primary analytical approach to evaluate treatment escalation dynamics.

**Table 2:** Comparison of Treatment Course According to ANA Status.

Treatment Parameter	ANA Positive (*n* = 112)	ANA Negative (*n* = 111)	*p*-Value
Number of csDMARDs used, median (IQR)	3.0 (2.0–3.0)	3.0 (2.0–3.0)	0.059
Failure of first csDMARD, *n* (%)	93 (83.0%)	99 (89.2%)	0.257
Transition to biologic therapy, *n* (%)	54 (48.2%)	27 (24.3%)	<0.001
Time to biologic therapy initiation, months (median (IQR))	41.0 (19.5–78.0)	26.0 (16.5–59.0)	0.630
Total number of biologics used, median (IQR)	1.0 (0.0–2.0)	0.0 (0.0–1.0)	0.004
Switching biologics ≥2, *n* (%)	33 (29.5%)	18 (16.2%)	0.028
First biologic class, *n* (%)			0.026
• Anti-TNF	40 (74.1%)	17 (63.0%)	
• IL-6 inhibitor (tocilizumab)	2 (3.7%)	7 (25.9%)	
• B-cell therapy (rituximab)	2 (3.7%)	0 (0.0%)	
• JAK inhibitor	2 (3.7%)	2 (7.4%)	
Duration of steroid use, months (median (IQR))	60.0 (36.0–100.0)	50.0 (26.8–96.0)	0.110
Mean daily steroid dose, mg (mean ± SD)	4.5 ± 1.0	4.5 ± 1.0	0.805
Cumulative steroid dose, mg (median (IQR))	288.0 (160–460)	240.0 (115–418)	0.093

Continuous variables were expressed as mean ± standard deviation (SD) or median [interquartile range (IQR)] according to distribution. Categorical variables were expressed as number (%). Comparisons were performed using Student’s *t*-test or Mann–Whitney U test for continuous variables and chi-square test or Fisher’s exact test for categorical variables. The *p*-value for first biologic class represents the overall comparison between groups (chi-square test). Distribution of first biologic classes was calculated among patients with available treatment classification data.

**Figure 1:**
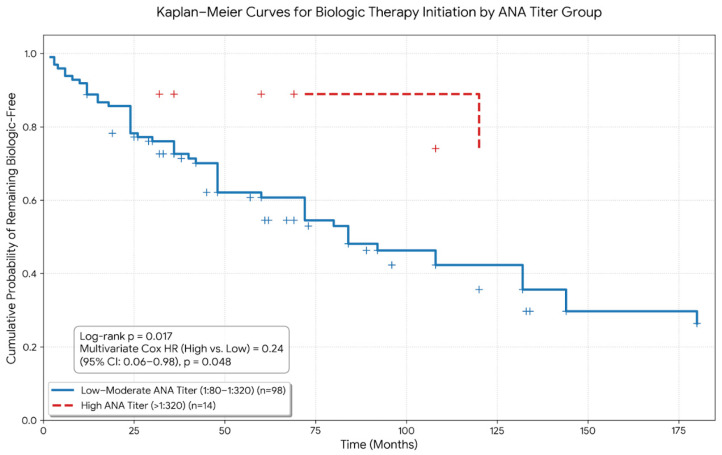
**Kaplan–Meier Curves for Biologic Therapy Initiation Stratified by ANA Titer Levels.** Kaplan–Meier survival curves illustrate the cumulative probability of remaining biologic-free over time in patients stratified by ANA titer groups: low–moderate ANA titer (1:80–1:320; *n* = 98, solid blue line) and high ANA titer (>1:320; *n* = 14, dashed red line). Censoring events are indicated by plus signs (+). The log-rank test demonstrates a significant difference between groups (*p* = 0.017). Multivariate Cox proportional hazards analysis shows that high ANA titer is associated with a decreased hazard of biologic therapy initiation compared to the low–moderate titer group (HR = 0.24, 95% CI: 0.06–0.98, *p* = 0.048).

Kaplan–Meier survival analysis demonstrated a significantly shorter time to biologic therapy initiation in ANA-positive patients compared with ANA-negative patients (log-rank *p* < 0.001; Figure 1). Patients who did not initiate biologic or targeted synthetic therapy during follow-up were censored at their last recorded clinical visit.

In univariate Cox regression analysis, ANA positivity was associated with an increased hazard of biologic therapy initiation. In addition, longer disease duration and lower baseline corticosteroid dose were also associated with a higher likelihood of initiating biologic therapy during follow-up.

**Figure 2:**
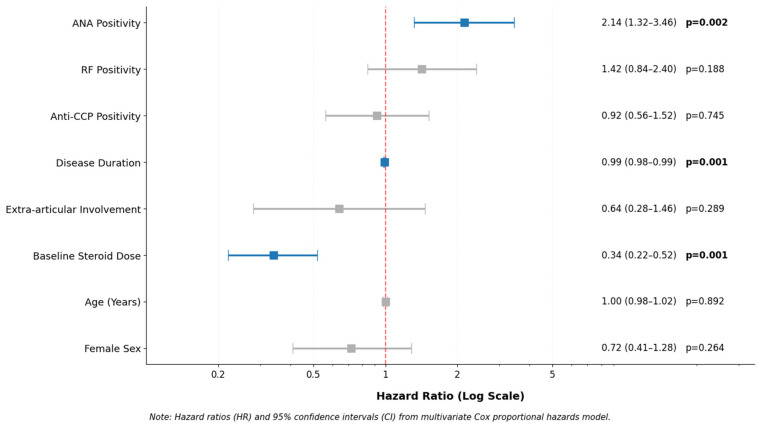
**Multivariate Cox Regression Analysis for Predictors of Biologic Therapy Initiation.** Forest plot showing hazard ratios (HR) with 95% confidence intervals (CI) for variables associated with time to biologic therapy initiation in the multivariate Cox proportional hazards model. Variables included are ANA positivity, RF positivity, Anti-CCP positivity, disease duration (months), extra-articular involvement, baseline corticosteroid dose (mg/day), age, and **female sex**. The vertical dashed red line represents HR = 1 (no effect). Statistically significant predictors (*p* < 0.05) are highlighted in bold. ANA positivity and lower baseline corticosteroid dose were associated with earlier biologic therapy initiation, whereas the per-month effect of disease duration was small (HR = 0.99) but may accumulate over longer periods.

Multivariate Cox proportional hazards analysis identified ANA positivity as an independent predictor of shorter time to biologic therapy initiation (adjusted HR: 2.14; 95% CI: 1.32–3.46; *p* = 0.002) after controlling for age, sex, disease duration, RF positivity, and anti-CCP positivity (Table 3, Figure 2).

RF and anti-CCP positivity were not independently associated with biologic therapy initiation (*p* = 0.188 and *p* = 0.457, respectively).

Additional independent predictors included longer disease duration and lower baseline corticosteroid dose, both of which were associated with a higher hazard of biologic therapy initiation during follow-up (both *p* < 0.001).

Within the ANA-positive cohort, 98 patients had low–moderate titers (1:80–1:320) and 14 patients had high titers (>1:320) (Supplementary Table S1). Baseline demographic and clinical characteristics were similar between titer subgroups. Given the small sample size of the high-titer group, these analyses are considered exploratory and hypothesis-generating. Kaplan–Meier survival analysis revealed a significant difference in time to biologic therapy initiation according to ANA titers (log-rank *p* = 0.017; Figure 1). Censoring events were defined as patients who did not initiate biologic therapy during follow-up and were last seen at their most recent clinic visit.

Interestingly, multivariate Cox regression indicated that high ANA titers were associated with a lower hazard of initiating biologic therapy compared with low–moderate titers (HR: 0.24; 95% CI: 0.06–0.98; *p* = 0.048). This illustrates a non-linear “titer paradox,” in which higher ANA concentrations did not necessarily correlate with more aggressive or synovial-dominant disease. These findings should be interpreted with caution due to limited events and subgroup size, and further studies are needed to confirm this unexpected association.

## 4. Discussion

In this retrospective cohort of patients with RA, ANA positivity was independently associated with shorter time to biologic therapy initiation after adjustment for conventional serological markers (RF and anti-CCP) and clinically relevant covariates. By defining time zero as the date of RA diagnosis and applying Cox proportional hazards modeling, we observed that ANA-positive patients experienced more rapid escalation to biologic therapy, suggesting a potential prognostic role of ANA beyond classical autoantibodies. However, these findings should be interpreted in the context of the retrospective design and potential confounding factors, such as physician practice patterns, comorbidities, and temporal changes in treatment availability. This observation suggests that ANA status may reflect immune processes associated with reduced responsiveness to conventional synthetic DMARDs, rather than being a mere epiphenomenon of autoimmunity. Further prospective studies are warranted to confirm this association and explore underlying immunophenotypic mechanisms.

Previous epidemiological studies have reported ANA positivity in RA cohorts with prevalence estimates ranging from 15% to 30%, although frequencies vary depending on patient selection and ANA detection methods, and clinical implications have remained inconsistent [9,15]. In our cohort, approximately 50% of patients were ANA-positive, which is higher than typically reported in the literature and may reflect the characteristics of our referral population or the serological testing methodology. Our findings align with recent real-world registry data indicating that ANA-positive RA patients, especially those exhibiting triple seropositivity (RF+/ACPA+/ANA+), demonstrate higher disease activity indices and reduced likelihood of sustained remission [19]. These patterns persisted even after accounting for RF and anti-CCP status, suggesting that ANA may provide independent prognostic information beyond classical markers. Nevertheless, the precise clinical relevance of ANA positivity remains exploratory and warrants confirmation in larger, prospective cohorts.

While RF and anti-CCP antibodies are well-established predictors of structural progression, they did not independently predict time to biologic therapy initiation in our multivariate models. This observation underscores that seropositivity alone may not consistently stratify risk for treatment escalation, particularly for targeted or biologic therapies. Prior work has similarly suggested that a broader autoantibody repertoire, rather than individual markers, correlates more strongly with refractory disease patterns [20,21]. These findings may reflect immunological heterogeneity within RA, where conventional serological profiles insufficiently capture complex B cell activation, epitope spreading, and other mechanisms driving chronic disease progression [22,23].

A particularly striking and novel finding in our study was the non-linear association between ANA titer and biologic therapy initiation. Although ANA positivity overall predicted earlier escalation, patients with high ANA titers (>1:320) were paradoxically associated with a lower hazard of biologic therapy initiation compared with those with low–moderate titers [24,25]. This “titer paradox” should be interpreted with caution due to the small sample size of the high-titer subgroup and borderline statistical significance, highlighting the exploratory nature of this analysis. Nonetheless, emerging immunophenotyping studies have suggested similar non-linear autoantibody effects, where high ANA titers may reflect systemic or type I interferon-enriched immunological signatures rather than synovial-dominant inflammation [26,27,28].

Mounting evidence highlights distinct molecular endotypes within RA, including type I interferon-dominant subgroups characterized by unique transcriptional profiles and distinct cytokine milieus. Such interferon-high RA endotypes may exhibit differential responses to biologic therapy compared with TNF-driven inflammation, where TNF inhibitors are typically more effective [29,30]. It is plausible and speculative that very high ANA concentrations may identify such interferon-enriched immunophenotypes, which could initially respond differently to conventional DMARDs and biologics. However, these mechanistic interpretations remain hypothetical, as our study did not include immunophenotypic or functional analyses.

Our observation associating lower baseline corticosteroid dose with earlier biologic therapy initiation likely reflects evolving clinical practices favoring steroid-sparing strategies and more aggressive treat-to-target approaches, rather than a direct biological effect of corticosteroids themselves. In line with recent European Alliance of Associations for Rheumatology (EULAR) recommendations advocating minimal long-term glucocorticoid use, clinicians may prefer earlier biologic intervention in patients with suboptimally controlled inflammation [31]. Nevertheless, residual confounding and physician-dependent decision-making cannot be excluded, and causal interpretations should be avoided.

Differences in first-line biologic selection between ANA groups were observed. These variations are likely attributable to clinician preference, temporal prescribing trends, or unmeasured patient- or disease-specific factors, rather than a direct mechanistic effect of ANA itself. Given the observational nature of our study and the lack of randomized allocation, causal inferences regarding ANA and specific biologic choice cannot be drawn.

This study has several limitations. The retrospective, single-center design introduces potential selection bias and may limit the generalizability of the findings. Despite verification of proportional hazards assumptions, residual confounding and unmeasured clinical factors cannot be entirely excluded. The exploratory titer analysis—especially in the high-titer subgroup—should be interpreted cautiously and warrants validation in larger, multicenter cohorts with comprehensive immunophenotyping.

Nevertheless, the study’s strengths include a robust time-to-event analytical framework, extended follow-up, systematic ANA titer stratification, and multivariate adjustment for established prognostic factors. Additionally, by evaluating both ANA positivity and titer levels, this study provides novel insights into potential immunophenotypic heterogeneity relevant to treatment escalation in RA.

Future research should prioritize mechanistic studies investigating the immunological basis of ANA-associated treatment refractoriness, particularly focusing on type I interferon pathways, B cell regulation, and autoantibody epitope expansion. Prospective, biomarker-driven clinical trials are needed to determine whether integrating ANA status—alone or in combination with multi-biomarker disease activity scores, ANA titer levels, and genomic risk profiles—can improve patient stratification, guide early treatment escalation, and enhance long-term outcomes. Such integrative precision medicine approaches may facilitate more individualized RA management and clarify the clinical relevance of ANA heterogeneity.

## 5. Conclusions

In summary, ANA positivity serves as an independent predictor of earlier and more intensive escalation to biologic therapy in patients with rheumatoid arthritis. This prognostic value appears to extend beyond traditional serological markers such as RF and anti-CCP antibodies, highlighting the potential utility of systematic ANA testing, including titer stratification, in informing individualized treatment strategies. Notably, the observed “titer paradox,” wherein high ANA levels were associated with delayed treatment escalation, underscores the complex, non-linear relationship between antibody concentration and therapy intensification, and warrants further mechanistic and immunophenotypic investigation. Collectively, these findings support the integration of comprehensive autoantibody profiling, encompassing ANA status and titer, into RA management frameworks to optimize early risk stratification, guide timely treatment escalation, and facilitate personalized therapeutic decision-making.

## Figures and Tables

**Table 1:** Baseline Demographic and Clinical Characteristics According to ANA Status.

Variable	ANA Positive (*n* = 112)	ANA Negative (*n* = 111)	*p*-Value
Age, years (mean ± SD)	54.2 ± 11.4	53.8 ± 12.1	0.812
Female sex, *n* (%)	94 (83.9%)	89 (79.5%)	0.384
Body mass index, kg/m^2^ (mean ± SD)	28.4 ± 4.2	27.9 ± 4.8	0.415
Disease duration, months (median (IQR))	114 (72–160)	108 (68–154)	0.542
Current smoker, *n* (%)	18 (16.1%)	22 (19.6%)	0.489
RF positivity, *n* (%)	78 (69.6%)	72 (64.3%)	0.395
Anti-CCP positivity, *n* (%)	64 (57.1%)	59 (52.7%)	0.504
ESR, mm/h (median (IQR))	32 (18–54)	28 (16–48)	0.124
CRP, mg/L (median (IQR))	12.4 (4.2–28.5)	10.8 (3.8–24.2)	0.231
DAS28 score (mean ± SD)	4.8 ± 1.2	4.6 ± 1.1	0.210
Hypertension, *n* (%)	24 (21.4%)	21 (18.8%)	0.612
Diabetes mellitus, *n* (%)	12 (10.7%)	14 (12.5%)	0.678
Cardiovascular disease, *n* (%)	8 (7.1%)	6 (5.4%)	0.582

Continuous variables were expressed as mean ± standard deviation (SD) or median [interquartile range (IQR)] according to distribution. Categorical variables were expressed as number (%). Comparisons were performed using Student’s *t*-test or Mann–Whitney U test for continuous variables and chi-square test for categorical variables. A *p*-value <0.05 was considered statistically significant.

**Table 3:** Univariate and Multivariate Cox Proportional Hazards Regression Analysis for Time to Biologic Therapy Initiation.

Variable	Univariate HR (95% CI)	*p*-Value	Multivariate HR (95% CI)	*p*-Value
ANA positivity	2.21 (1.41–3.48)	<0.001	2.14 (1.32–3.46)	0.002
RF positivity	1.84 (1.12–3.02)	0.016	1.42 (0.84–2.40)	0.188
Anti-CCP positivity	1.15 (0.74–1.78)	0.532	0.92 (0.56–1.52)	0.745
Disease duration (months)	0.99 (0.98–1.00)	0.001	0.99 (0.98–0.99)	<0.001
Extra-articular involvement	0.52 (0.24–1.12)	0.094	0.64 (0.28–1.46)	0.289
Baseline steroid dose (mg/day)	0.35 (0.23–0.54)	<0.001	0.34 (0.22–0.52)	<0.001
Age (years)	0.99 (0.97–1.01)	0.245	1.00 (0.98–1.02)	0.892
Female sex	0.78 (0.45–1.34)	0.364	0.72 (0.41–1.28)	0.264

All 223 patients from the cohort were included in the Cox regression analysis. Hazard ratios (HR) were calculated using Cox proportional hazards regression. Variables with *p* < 0.10 in univariate analysis were included in the multivariate model. Continuous variables are expressed per unit increase. For disease duration, hazard ratios correspond to the effect per additional month of disease duration.

## Data Availability

The original contributions presented in this study are included in the article/Supplementary Material. Further inquiries can be directed to the corresponding authors.

## Supplementary Materials

The following supporting information can be downloaded at: https://www.mdpi.com/article/10.3390/diagnostics16060957/s1, Supplementary Table S1 presents baseline characteristics of ANA-positive patients stratified by titer level. Comparisons were conducted using non-parametric and exact tests due to the small size of the high-titer subgroup (*n* = 14). This exploratory analysis should be interpreted cautiously.

## Abbreviations

ACR/EULAR	American College of Rheumatology/European Alliance of Associations for Rheumatology
ANA	Antinuclear Antibody
Anti CCP	Anti Cyclic Citrulinated Protein
DMARD	Disease Modifying Antirheumatic Drug
bDMARD	Biologic Disease-Modifying Antirheumatic Drug
tsDMARD	Targeted Synthetic Disease-Modifying Antirheumatic Drug
csDMARD	Conventional synthetic Disease-Modifying Antirheumatic Drug
BMI	Body Mass Index
CRP	C-Reactive Protein
DAS28	Disease Activity Score in 28 joints
ESR	Erythrocyte Sedimentation Rate
IIF	Indirect Immunofluorescence
IL-6	Interleukin-6
JAK	Janus Kinase
RF	Rheumatoid Factor
RA	Rheumatoid Arthritis
