# Supplementary material for: Antinuclear Antibodies Predict Treatment Escalation and Biologic Switching in Rheumatoid Arthritis
Source: Diagnostics (Basel). 2026 Mar 23;16(6):957. doi: 10.3390/diagnostics16060957 (PMC13026019; doi:10.3390/diagnostics16060957)
Supplement: Supplementary file 1 [file diagnostics-16-00957-s001.zip › diagnostics-4191905-supplementary.pdf]

Supplementary Table S1. Baseline Demographic and Clinical Characteristics According to ANA Titer Levels (Exploratory Analysis, n=112)

**Variable**	**Low–Moderate ANA (1:80–1:320) (n=98)**	**High ANA (>1:320) (n=14)**	**p-value**
Age, years (mean ± SD)	50.0 ± 12.5	50.6 ± 14.3	0.862
Female sex, n (%)	85 (86.7%)	10 (71.4%)	0.274
Disease duration, months (median [IQR])	84.0 [48.0–141.5]	108.0 [62.2–153.5]	0.441
ESR (mm/h) (median [IQR])	16.0 [7.0–25.0]	24.5 [12.2–33.5]	0.066
CRP (mg/L) (median [IQR])	6.4 [2.2–14.5]	8.9 [2.8–12.7]	0.778
RF positivity, n (%)	82 (83.7%)	9 (64.3%)	0.170
Anti-CCP positivity, n (%)	61 (62.2%)	9 (64.3%)	1.000

Data are presented as mean ± standard deviation (SD) for normally distributed variables or median [interquartile range (IQR)] for non-normally distributed variables. Categorical variables are expressed as number (%). Comparisons were performed using Mann–Whitney U test for continuous variables and Fisher's exact test for categorical variables due to the small sample size in the high-titer group. This analysis is exploratory, and results should be interpreted with caution given the small number of patients in the high-titer subgroup (n=14).
